# Fabry’s Disease: The Utility of a Multidisciplinary Screening Approach

**DOI:** 10.3390/life12050623

**Published:** 2022-04-22

**Authors:** Marco Angelo Monte, Massimiliano Veroux, Margherita Stefania Rodolico, Valentina Losi, Luigi Di Pino, Rita Bella, Giuseppe Lanza, Ines Paola Monte

**Affiliations:** 1 Department of Surgery and Medical-Surgical Specialties, University of Catania, Via Santa Sofia 78, 95123 Catania, Italy; marcom.159@hotmail.it (M.A.M.); vale.losi@gmail.com (V.L.); dipino@unict.it (L.D.P.); giuseppe.lanza1@unict.it (G.L.); 2 Department of Medical and Surgical Sciences and Advanced Technologies, University of Catania, Via Santa Sofia 78, 95123 Catania, Italy; veroux@unict.it (M.V.); rbella@unict.it (R.B.); 3 C.N.R. Institute for Biomedical Research and Innovation—IRIB, Via P. Gaifami 18, 95126 Catania, Italy; margheritastefania.rodolico@cnr.it; 4 Clinical Neurophysiology Research Unit, Oasi Research Institute-IRCCS, Via Conte Ruggero 73, 94018 Troina, Italy

**Keywords:** Fabry’s disease, lysosomal storage disease, genetics, multidisciplinary, screening

## Abstract

(1) Background: As a lysosomal storage disorder, Fabry’s disease (FD) shows variable clinical manifestations. We applied our multidisciplinary approach to identify any organ damage in a sample of adult patients with different pathogenic variants. (2) Methods: 49 participants (mean age 44.3 ± 14.2 years; 37 females), underwent a multidimensional clinical and instrumental assessment. (3) Results: At diagnosis, mean enzymatic activity was 5.2 ± 4.6 nM/mL/h in females and 1.4 ± 0.5 nM/mL/h in males (normal values > 3.0), whereas globotriaosylsphingosine was 2.3 ± 2.1 nM/L in females and 28.7 ± 3.5 nM/L in males (normal values < 2.0). Overall, cardiovascular, neurological, and audiological systems were the most involved, regardless of the variant detected. Patients with classic variants (10) showed typical multiorgan involvement and, in some cases, prevalent organ damage (cardiovascular, neurological, renal, and ocular). Those with late-onset variants (39) exhibited lower occurrence of multiorgan impairment, although some of them affected the cardiovascular and neurological systems more. In patients with lower enzymatic activity, the most frequent involvement was neurological, followed by peripheral vascular disease. (4) Conclusions: FD patients exhibited wide phenotypic variability, even at single-organ level, likely due to the individual genetic mutation, although other factors may contribute. Compared to the conventional management, a multidisciplinary approach, as that prompted at our Center, allows one to achieve early clinical detection and management.

## 1. Introduction

### 1.1. Background

Fabry’s disease (FD) is an X-linked lysosomal storage disorder caused by pathogenic variants in the α-galactosidase A (α-GAL-A) (*GLA*) gene, which lead to a reduced or absent lysosomal α-GAL-A enzymatic activity, thus, impairing lysosomal functioning [1]. This causes a progressive accumulation of glycosphingolipids, primarily globotriaosylceramide (Gb3) and its deacylated form globotriaosylsphingosine (lyso-Gb3), in cells and tissues, such as smooth muscle cells, vascular endothelium, cardiomyocytes, and kidneys [2]. The increasing prevalence of *GLA* gene mutations, from previous estimates of 1:40,000–170,000 [3] up to 1:1250 in newborn screening studies [4,5,6], reflects the existence of a majority of non-classic mutations and variants of unknown significance (VUS), where natural history and effectiveness of enzyme replacement therapy (ERT) are still unknown [7]. Neurological, cardiological, and nephrological alterations, as well as their complications, represent the main causes of morbidity, mortality, and disability in these patients [8].

As a systemic disease, FD may show a spectrum of clinical manifestations, such as cardiological, renal, neurological, ocular, dermatological, and gastrointestinal features [9,10]. As such, patients require a comprehensive assessment for both diagnostic and therapeutic purposes, with a “patient-centered” model of care. Different studies, indeed, have highlighted the importance of a multidisciplinary team in the early evaluation, management, and follow-up of both adults and pediatric FD patients [11]. However, the clinical variability of different mutations, as well as variability in disease severity and symptom onset, make the diagnosis often challenging. There is, indeed, a wide clinical heterogeneity, even within the same family, and large intersex variability [12]. Moreover, more than 900 variants of the *GLA* gene have been identified [13], many of them being VUS, and, in these cases, invasive diagnosis investigating the presence of tissue Gb3 deposits may be indicated. Therefore, there is a need for a reliable diagnostic approach and biomarkers [14,15].

In this scenario, in our previous study [16], patients with pathogenic *GLA* variants, among 265 kidney transplant recipients, underwent complete cardiovascular, nephrological, neurologic, ophthalmologic, dermatologic, gastroenterological, endocrinological, pneumological, and psychiatric evaluations, thus, allowing early diagnosis and adequate management of any organ-related clinical manifestations of FD. Regarding treatment and follow-up, patients should be supervised by a physician experienced in FD, within a team of highly experienced specialists, including a cardiologist, neurologist, nephrologist, ophthalmologist, dermatologist, geneticist, psychologist, and nurse, among others [17]. 

### 1.2. The Fabry Multidisciplinary Research Center

In 2008, the University Hospital of Catania (Italy) established a multidisciplinary team with a specific expertise in FD, called “Fabry Multidisciplinary Research Center” (FMRC), which encompasses more than 30 different specialists, dealing with the screening, assessment, diagnosis, management, treatment, and follow-up of patients with pathogenic *GLA* variants. The FMRC has also provided the optimal framework to design and carry out scientific activities on FD [16]. Of note, all the specialists involved work within the same hospital, thus, avoiding the need for multiple, time-consuming, and costly visits to other centers. The FMRC evaluates both patients and families, performs a comprehensive assessment of any diagnosed or suspected patient, detects new patients or suspicious cases (even preclinically), and reduces the delay between the first consultation and the diagnosis, which is normally completed within a mean time of 3.2 months and with a mean time from diagnosis to treatment of 4.6 months. A timely diagnosis allows for a prompt treatment, before irreversible organ damage occurs, and this is associated with better long-term outcomes. After being diagnosed, all subjects are included in an institutional database and undergo genetic counselling and family screening, whereas their management and treatment basically depend on different factors. Namely, asymptomatic subjects, as well as those without relevant signs or symptoms, clear alterations at the instrumental exams, any significant systemic or organ damage, and functionally independent or with an acceptable quality of life, are usually followed on a yearly basis. Conversely, patients clinically affected, especially if cardiovascular, renal, or neurological signs are present, are evaluated for ERT, according to the consensus guidelines [18,19]. 

Briefly, classically affected males, classically affected females, and males with non-classical FD are normally treated as soon as early clinical signs of kidney, heart, or brain involvement occur, whereas the possibility of treatment is considered in females with non-classical FD but early clinical signs of FD. Conversely, we usually stop ERT in patients with end-stage FD or other co-morbidities leading to a life expectancy lower than 1 year, as well as in non-compliant subjects or in those failing to regularly attend the visits. In those with cognitive decline of any cause or lack of response for at least one year when the sole indication for ERT is neuropathic pain, as well as in those with end-stage renal disease (ESRD), without options for renal transplantation, and in combination with advanced heart failure, we consider cessation of ERT [18,19]. In other types of patients or in any complex or doubtful case, ERT is carefully considered on an individual basis.

At the regional level, this approach has also led to the definition of the so-called “Diagnostic Therapeutic Care Path” (in Italian, “Percorso Diagnostico Terapeutico Assistenziale”, PDTA). PDTAs are standardized clinical procedures and management guidelines to apply in a local context and specific disease. They are established according to national and/or international guidelines, recommendations, and consensus, aiming at defining the best clinical and healthcare practice to fulfill the patients’ need [20]. Every PDTA has to: (i) comply with the clinical and diagnostic needs of the specialists dealing with the disease of interest (Disease Management); (ii) constantly monitor and improve the network in order to achieve and maintain the best standard of care (Clinical Governance); (iii) train and support all the operators involved in each step of the path, including the optimal timing and equity of access for the population of interest (Public Management); (iv) organize and take care of the all the structures and dedicated facilities (Operating Units).

As such, the diagnosis of FD needs a complex clinical, biochemical, and genetic evaluation, from a detailed personal and family history (including the reconstruction of the family tree) to an accurate clinical examination, and from a number of laboratory tests to different instrumental exams [21]. Diagnosis in hemizygous males is based on the demonstration of enzyme deficiency in leukocytes or fibroblasts, whereas the genetic test allows one to analyze and confirm the culprit pathogenetic variant. As known, however, in heterozygous females, the enzymatic dosage may not be conclusive, because of the “mosaic effect” due to the X-chromosome lyonization; therefore, genetic testing is necessary [22,23]. Based on these considerations, the diagnostic path of FD should include: (i) a Point of Access, often through the general practitioner/pediatrician or various medical specialists; (ii) Specialist Spoke Center, who performs the first-line examinations and, in case of clinical suspicion, requests confirmation to the regional Hub Center; (iii) Specialist Hub Center, which, based on the regional PDTA, diagnoses and defines the therapeutic choices [17]. 

In our region, this path consists of two steps:( i) presumptive diagnosis and disease investigation and (ii) definitive diagnosis and disease certification [16]. In the first step, every specialist within the FMRC can suspect FD based on personal and family history and suggestive clinical signs and symptoms. The patient is, therefore, included in the PDTA through a regional inter-hospital network (Spoke Centers) or, in case of strong suspicion, directly referred to the Hub Center. In the second step, the definitive diagnosis is obtained through the genetic test, which also allows for the identification of any pathogenetic variant. The Hub Center also provides disease certification, detailed counselling, and therapeutic prescriptions, as well as the timing and modality of follow-up visits [16]. Additionally, in every Spoke Center, the PDTA identifies a highly experienced specialist (the so-called “case manager”), who coordinates all clinical activities, basically based on the patient’s age. For pediatric patients, the case manager is often a pediatrician with expertise in FD, whereas for adult subjects, the case manager can be a specialist of different clinical disciplines, although this is often an internal medicine physician or a cardiologist [16,23,24].

### 1.3. Aim

In this study, we aimed to apply the PDTA-based multidisciplinary approach of our FMRC to comprehensively assess and identify the presence of any organ damage early, in a group of adult patients with different types of pathogenic FD variants.

## 2. Materials and Methods

In the present study, 49 participants (37 females, 12 males), aged >18 years (mean age ± standard deviation: 44.3 ± 14.2 years old), recruited by the Cardiology Department of the University Hospital of Catania between 2009 and 2021 and managed by the FMRC, were included. In addition to the high prevalence of cardiological manifestations in FD (especially in the late-onset phenotype [25], as in the present study), as well as the need for a cardiovascular screening in patients’ relatives and in any suspected case, the Cardiology Department of the University Hospital of Catania (namely, the Center for Rare Cardiomyopathies) was identified as the Regional Centre for FD. 

FD diagnosis was performed with the genetic analysis for pathogenic *GLA* variants analyzed through the polymerase chain reaction in all participants from peripheral blood sample, as recommended [24,25]. Analyses of the *GLA* gene, lyso-Gb3 levels, and α-Gal-A enzymatic activity were performed at Centogene^©^ Laboratories (Rostok, Germany). *GLA* variants were classified according to American College of Medical Genetics and Genomics recommendations [26], in: (i) pathogenic variants: a well-established disease cause, with a strong genotype–phenotype correlation; (ii) likely pathogenic variants: a probable cause of the patient’s phenotype; (iii) VUS: a genetic variant with unknown or questionable impact on clinical phenotype; (iv) likely benign: a variant with low probability of causing the disease or phenotype; (v) benign: a variant without clinical significance.

For each subject, all symptoms and signs associated with genetically defined FD were included in the above-mentioned institutional database, whereas those observed in subjects with VUS and benign or likely benign variants were excluded [27]. Regarding the multidisciplinary assessment at the time of diagnosis, several laboratory and instrumental exams were performed, in order to comprehensively define the occurrence, extent, and monitoring of different organ involvement (Table 1). In case of unknown disease in the family but with strong suspicion for FD, as well as in first examined patients, an anamnestic survey was administered in order to screen any related symptom or disorder possibly associated with FD. Annual follow-up was scheduled for asymptomatic patients or those without clear alterations at imaging; conversely, patients with signs or symptoms of FD (e.g., stroke, chronic kidney disease, hypertrophic cardiomyopathy, cornea verticillate, etc.) were evaluated for ERT, as recommended [22,23,24,25,28].

Sixteen patients were on FD-specific treatment, namely: migalastat (2 females and 3 males), agalsidase alfa (4 females and 4 males), and agalsidase beta (3 females). 

The study was conducted in accordance with the Declaration of Helsinki and approved by the Ethics Committee of the Azienda Ospedaliero-Universitaria Policlinico “G. Rodolico-San Marco” of Catania, Italy (protocol code: 0004003; approval date: 28 January 2019). Written informed consent was obtained from all subjects involved in the study.

## 3. Results

At diagnosis, mean enzymatic activity was 5.2 ± 4.6 nM/mL/h in females and 1.4 ± 0.5 nM/mL/h in males (normal values > 3.0), whereas the lyso-Gb3 value was 2.3 ± 2.1 nM/L in females and 28.7 ± 3.5 nM/L in males (normal values < 2.0). Table 2 summarizes all the *GLA* variants detected. A pathogenic variant, classic or late-onset, was identified in 10 and 39 subjects, respectively. According to the type of variant, mean enzymatic activities were 5.7 ± 4.9 nM/mL/h in females and 0.6 ± 0.5 nM/mL/h in males, in patients with a late-onset pathogenetic variant, whereas they were 3.6 ± 3.1 nM/mL/h in females and 0.4 ± 0.3 nM/mL/h in males, in those with a classic pathogenetic variant. Regarding lyso-Gb3 values, they were 1.9 ± 1.1 nM/L in females and 4.5 ± 4.4 nM/L in males, in patients with a late-onset pathogenetic variant and 8.7 ± 3.9 nM/L in females and 65.2 ± 18.3 nM/L in males, in those with a classic pathogenetic variant.

**Figure 1:**
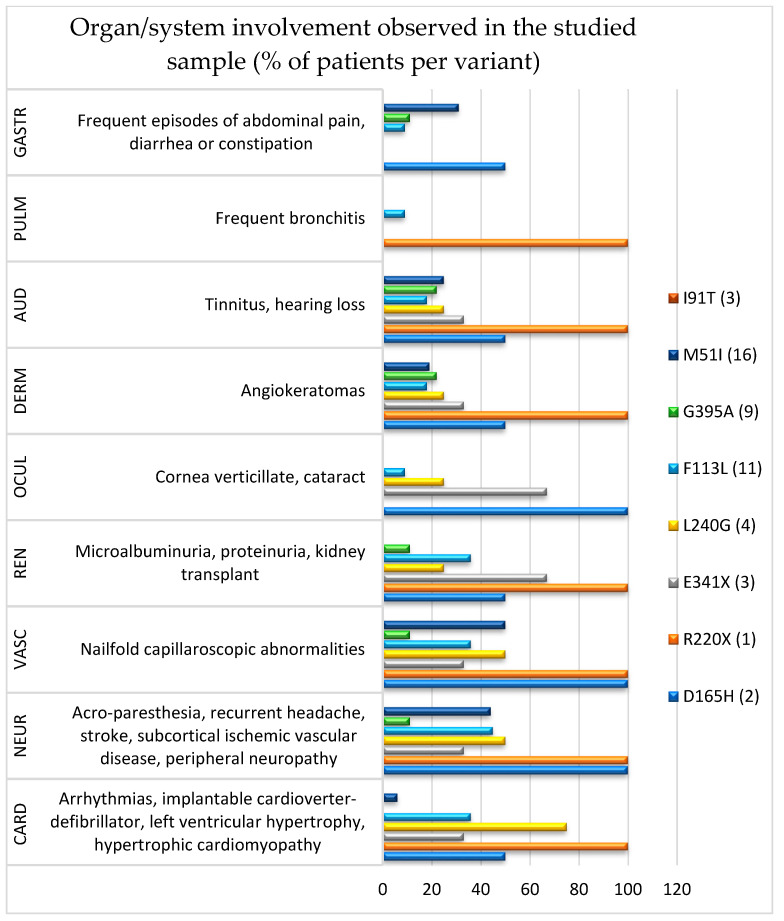
Organ/system involvement observed in the studied sample and variants. GASTR, gastrointestinal; PULM, pulmonary; AUD, audiological; DERM, dermatological; OCUL, ocular; REN, renal; VASC, vascular; NEUR, neurological; CARD, cardiac involvement.

Based on the anamnestic survey and clinical–diagnostic evaluation, multiple-organ involvement was detected in most of the patients (Figure 1). Globally, cardiovascular (61.2%) and neurological (38.8%) systems were the most affected, followed by audiological (24.5%), renal (20.4%), dermatological (20.4%), gastrointestinal (16.3%), ocular (12.2%), and pulmonary (4.1%), regardless of the variant detected. Overall, patients with classic pathogenic variants (10) showed the typical multiorgan involvement and, in some cases, prevalent organ damage, i.e., cardiovascular (60.0%), neurological (60.0%), renal (50.0%), and ocular (50.0%), followed by audiological (40.0%), dermatological (30.0%), pulmonary (10.0%), and gastrointestinal (10.0%). Those with late-onset pathogenic variants (39) exhibited a lower occurrence of multiorgan impairment, although some of them were more affected in the cardiovascular (46.1%) and neurological systems (33.3%), followed by audiological (20.5%), gastrointestinal (17.9%), dermatological (17.9%), renal (12.8%), pulmonary (2.6%), and ocular (2.6%). 

Clinically, patients with the classic pathogenetic variant L240G showed prevalent cardiovascular and neurological damage, whereas more prominent renal and ocular involvement was noted in those with E341X. In the two patients with the D165H variant, vascular, neurological, and ocular systems were all involved, whereas diffuse manifestations (except for ocular, dermatological, and gastrointestinal districts) were found in the only subject with the R220X variant. Although patients with late-onset pathogenetic variants exhibited a lower occurrence of multiorgan damage, the F113L and M51I variants affected almost all the systems explored. Conversely, in the other variants (G395A and I91T), both diffuse involvement and single-organ manifestations were less common. Finally, in patients with lower enzymatic activity (<3 nmol/mL/h), the most frequent involvement was neurological, followed by peripheral vascular disease. Nineteen patients were followed during the last two years, although in 2020, the follow-up was stopped due to the COVID-19 pandemic.

## 4. Discussion

FD is a rare, progressive, and life-threatening lysosomal storage disease, whose natural history can be significantly modified by ERT. In the present study, our PDTA-based multidisciplinary approach showed that FD patients exhibited wide phenotypic variability, even at the single-organ level. Therefore, early diagnosis and prompt management are the optimal target for these patients but, concomitantly, a challenge [29,39]. On one hand, we confirmed the manifestations of the organs/systems typically involved in FD (i.e., cardiovascular, renal, neurological, and dermatological); on the other hand, however, we also demonstrated a relatively frequent involvement of other systems (i.e., audiological, ocular, gastrointestinal, and pulmonary), regardless of the variant detected.

In this context, international guidelines for the recognition, assessment, surveillance, and therapy (including ERT) have been proposed to optimize patients’ outcomes [40]. The multidisciplinary approach has also been demonstrated to provide better outcomes in terms of disease stability after ERT and clinical regression, including patients with mild organ impairment [41]. Finally, a very recent study in a Chinese hospital highlighted that the multidisciplinary assessment allowed for the identification of FD in 35 high-risk children, whereas none were identified before the establishment of the multidisciplinary team [42].

Overall, it should be mentioned that there was no predominant organ damage with respect to the others in the present population of FD. This might be explained by the evidence that organ involvement mainly depends on the pathogenic *GLA* variant identified [43,44,45], although other factors might contribute, such as genetic variability, even within the same parental nucleus. For instance, even neurologically asymptomatic patients may show imaging or neurosonological evidence of cerebrovascular disease, mainly affecting the posterior circulation, as recently demonstrated [46,47]. Therefore, preclinical detection of neurovascular involvement in FD might help to prevent future cerebrovascular complications and related motor and cognitive disability.

Among the classic phenotypes, the pathogenetic variant D165H has been associated with typical clinical features (e.g., cornea verticillate, angiokeratomas, ESRD, cardiological and neurological manifestations) [48]. Additionally, we consistently found other manifestations, involving audiological, pulmonary, and gastrointestinal systems, thus, possibly expanding the clinical spectrum of D165H mutations. Even more interestingly, although the R220X variant is known to cause typical FD in both genders (especially affecting the heart and kidney [49]), we observed that all the organs/systems explored in this study were involved. However, this mutation was detected in one subject only and, therefore, further genotype–phenotype correlations are needed. 

Little is known also on the E341X and L240G variants, which have been linked to classic FD, i.e., cornea verticillata, hypo-anhidrosis, left ventricular hypertrophy, cerebrovascular diseases, and renal failure [50], which have been globally observed in our patients as well. Nevertheless, one-third of those with the E341X variant also showed peripheral vascular, dermatological, and audiological impairment, whereas 50% and 25% of those carrying the L240G variant also had peripheral vascular and audiological damage, respectively. 

Regarding late-onset pathogenetic variants, our findings seem to be in line with the literature. Namely, the F113L mutation is known to be constantly associated with cardiac damage, along with clinically relevant cerebrovascular and kidney disease in some patients [51]. Accordingly, in our F113L carriers, the occurrence of cardiovascular, neurological, and renal damage was relatively high; conversely, a lower rate of dermatological, audiological and, even less, ocular, pulmonary, and gastrointestinal disease was noted. Therefore, these data deserve further investigation, as well as for the G395A variant. 

The wide variability in FD manifestations is also supported by the M51I variant, which correlates with atypical phenotypes in terms of organs involved and severity, as previously described in an extended Italian family [52]. Coherently, half of our patients with this mutation showed peripheral vascular disease, followed by gastrointestinal, audiological, and dermatological, whereas typical features (i.e., cardiac, renal, and ocular) were much less frequent. Lastly, the I91T variant has been recently associated with ESRD [53], as also observed in our patients. 

Translationally, a multisystemic approach, such as that provided by our FMRC, may help to appropriately evaluate these variants, even in case of low clinical significance in the literature, and contribute to expanding the spectrum of both systemic and organ-related manifestations of FD [5,39]. In this context, it is worth mentioning that the prominent involvement of the peripheral vascular system may be conditioned by the high diagnostic hints provided by the nailfold capillaroscopy, which is carried out in most FD patients (including those in the present study). On the other hand, this supports the concept of vascular inflammation and endothelial dysfunction in FD [54] and their translational implications in the treatment of these patients [23,54,55].

The strengths of this study are the inclusion of an adequate sample of subjects with a rare disease, the possibility to correlate a number of clinical districts with different types of pathogenic variants, and the approach based on our experience in FD, which has lasted for more than 15 years, 5 of which have already passed since the birth of the FMRC. 

The main limitations are the single-center design, the limited number of patients with classic pathogenic variants, the inclusion of adult patients only and with predominantly females [56], as well as the loss of many patients at follow-up. The possibility to remotely follow-up these patients was limited (e.g., through telemedicine or similar tools), except for clinical interviews and limited aspects of some (e.g., dermatological) examinations, as all clinical evaluations and instrumental exams included in this study needed to be physically carried out. In the pandemic scenario, however, restrictions and other organizational difficulties considerably limited this part of the study. Crucial research questions regard the possibility to develop a diagnostic tool or an *ad hoc* score, able to identify non-pathogenic mutations, and to provide reliable genotype–phenotype correlations.

## 5. Conclusions

The development and application of a multidisciplinary team and a specific path for FD patients, as with the FMRC prompted at our Center and applied through the regional PDTA, allows for the early diagnosis and selection of patients to treat and strictly follow-up. This approach also helped to differentiate patients with pathogenic variants from those individuals with VUS or benign/likely benign variants. Ideally, such an integrated model of care should be part of routine medical practice, not only for FD, but also for similar disorders. Reducing fragmentation in healthcare and promoting “patient-centered” care is necessary in rare medicine and needs to be further promoted.

## Figures and Tables

**Table 1:** Main laboratory and instrumental exams to assess the organ involvement in FD patients.

Organ/System	Assessment
Cardiological [23,28,29]	Blood pressure monitoring and electrocardiogram (ECG) at rest Dynamic ECG, including the heart rate variability analysis Echocardiogram, with tissue Doppler and global longitudinal strain Maximum ergometric test Cardiac magnetic resonance imaging (MRI), with different evaluation criteria for males and females
Neurological [30]	Brain MRI to display lacunar lesions or leukoencephalopathy Angio-MRI or angio-computed tomography (CT) scan Electromyography and electroneurography Multimodal evoked potentials Autonomic nervous system evaluation
Audiological [31]	Tonal audiometry Brainstem auditory evoked potentials
Renal [16,32]	Serum creatinine (with glomerular filtration rate), urea, electrolytes Urinary sediment to display typical cytoplasmic within excreted tubular cells, albuminuria, proteinuria, and assessment with polarized light to reveal the typical “crosses of Malta” due to lipiduria Renal ultrasound to assess size and morphology In selected cases, renal biopsy and optical and electronic microscopy
Ocular [33]	Assessment of cornea verticillate and FD-related cataract Ophthalmoscopic evaluation
Peripheral vascular [34,35]	Angiological evaluation Peripheral vascular Doppler ultrasound Nailfold capillaroscopy
Pulmonary [36]	Assessment of obstructive airway limitation (chest X-ray, CT) Spirometry
Gastrointestinal [37]	Assessment of gastrointestinal simptoms Abdomen ultrasound and, in selected case, CT scan and endoscopy
Dermatological [38]	Identification and characterization of typical angiokeratomas and other cutaneous manifestations

**Table 2:** Detected *GLA* variants (in brackets, the number of patients with the variant).

Type of Variant	Detected Variant (Number of Patients)
Pathogenetic (classic)	D165H (2), R220X (1), E341X (3), L240G (4)
Pathogenetic (late-onset)	F113L (11), G395A (9), M51I (16) I91T (3)

## Data Availability

The data presented in this study are available on request from the corresponding author.
